# A new species of *Brevianthus* (Brevianthaceae, Marchantiophyta) from New Caledonia with unusual underleaf production

**DOI:** 10.3897/phytokeys.50.4998

**Published:** 2015-06-04

**Authors:** Matt A.M. Renner, John J. Engel, Simon D.F. Patzak, Jochen Heinrichs

**Affiliations:** 1 Royal Botanic Gardens & Domain Trust, Mrs Macquaries Road, Sydney, NSW 2000, Australia; 2 The Field Museum, 1400 S Lake Shore Drive, Chicago IL 60605-2496, USA; 3 Systematische Botanik und Mykologie, Ludwig-Maximilians-Universität München, 80638 München, Germany

**Keywords:** Bryophyte, liverwort, *Brevianthus flavus*, New Zealand, Tasmania, Australasia

## Abstract

*Brevianthus* is a distinctive genus of leafy liverwort in its succubously inserted, entire leaves, lack of underleaves, restriction of sexual organs to lateral-intercalary branches, scattered rhizoids and dense leaf-surface ornamentation. The sole species, *Brevianthus flavus*, is divided into two subspecies, one in Tasmania the other in New Zealand. A second species, *Brevianthus hypocanthidium*, is described as new and is the first record of the genus for New Caledonia. Among its distinguishing characters are its shallowly bilobed leaves, and triangular underleaves present on small to medium-sized shoot sectors, the lack of a hyaline leaf margin, and the crenulate leaf margin formed by heavily thickened external cell walls. The most unusual features of the new species are the presence of underleaves between lateral leaf insertion lines that reach the ventral stem mid-line, and the absence of underleaves from larger shoots. To explain these features we propose a competitive model of shoot formation wherein the ventral merophyte progressively loses vigor as its relative stature decreases, and its derivative cells become discontinuous and isolated along the ventral stem surface, with intervening areas occupied by derivatives of the more vigorous lateral merophytes.

## Introduction

*Brevianthus* J.J.Engel & R.M.Schust. possesses a highly distinctive suite of morphological characters incongruent with its phylogenetic relationships. The restriction of sexual organs to abbreviated lateral-intercalary branches, the lack of underleaves, and the scattered rhizoids were thought to immediately remove the plant from the Lophocoleaceae De Not. (Engel and Schuster 1982), whereas the combination of succubously inserted, undivided leaves, with coarse trigones, lack of underleaves, undifferentiated stems, and short androecial branches suggest the isolated genus *Jackiella* Schiffn., within which the type species was originally described by Grolle (1970) on the basis of sterile and androecial individuals. The discovery of fertile, perianth- and sporophyte-bearing material prompted the species’ removal from Jackiellaceae R.M.Schust. due to the well-developed perianth and the comparatively large and undifferentiated seta. The female structures in combination with the minute androecia with strongly ventricose and monandrous bracts, the uniseriate antheridial stalks suggested relationships with the Adelanthaceae Grolle. However, the exclusively lateral intercalary branching (both vegetative and sexual), lack of stolons, lack of secondary pigmentation, and the 3-4 stratose capsule were all inconsistent with placement within the Adelanthaceae (Engel and Schuster 1982).

The wide-mouthed obscurely trilobed perianths, the isophyllous gynoecium, the 1-phase development of the outer capsule layer, and the seta anatomy all suggested affinity with the old Geocalycineae R.M.Schust. (including the Lophocoleaceae and Plagiochilaceae Müll. Frib. & Herzog). However the spherical capsules, scattered rhizoids and apparent absence of a ventral merophyte were anomalous with that suborder so *Brevianthus* was placed, with the Chonecoleaceae R.M.Schust. ex Grolle, into an independent new suborder, Brevianthineae J.J.Engel & R.M.Schust. by Engel and Schuster (1982). These authors also proposed a monogeneric family Brevianthaceae J.J.Engel & R.M.Schust.

Molecular phylogenetic studies led to considerable changes in the classification of liverworts (Crandall-Stotler et al. 2009) and supported the reinstatement of Lophocoleaceae (Hentschel et al. 2007) for the perianth-bearing elements of Geocalycaceae Endl. sensu Crandall-Stotler and Stotler (2000) and others.

The first molecular phylogenetic study including *Brevianthus*, on the basis of a New Zealand specimen, resolved the genus sister to the Lophocoleaceae element *Tetracymbaliella* Grolle, then *Plagiochila* (Dumort.) Dumort. in a monophylum also containing *Chiloscyphus* Corda (He-Nygren et al. 2006), placing it firmly within the Lophocoleaceae-Plagiochilaceae familial complex in contradiction to much of the morphological evidence. Subsequent molecular phylogenetic studies seem to corroborate the sister relationship of *Brevianthus* and *Tetracymbaliella*, and resolved this clade as sister to the rest of the Lophocoleaceae (Feldberg et al. 2014).

Once believed endemic to Tasmania, *Brevianthus* was discovered on South Island (Blackball), New Zealand in 1998 (Glenny 2000), and has since been collected from a small number of sites on the West Coast of the South Island and in the upper North Island. The first New Zealand collection was made in the North Island in 1990 but went unrecognized. New Zealand plants differ morphologically from Tasmanian, and were given subspecific status by Engel (2011).

In 2006 the late Elizabeth Brown made a relatively copious collection of *Brevianthus* at Mont Kouakoué in New Caledonia, sufficient to facilitate the identification of several distinctive morphological characters warranting its assignment as a separate species. We outline this proposition below, and provide additional observations on the ecology and distribution of the two subspecies of *Brevianthus flavus* (Grolle) J.J.Engel & R.M.Schust.

## Taxonomic treatment

### Brevianthus

Taxon classificationPlantaeJungermannialesBrevianthaceae

J.J.Engel & R.M.Schust., Phytologia 47: 317. 1981.

#### Type.

*Brevianthus flavus* (Grolle) J.J.Engel & R.M.Schust., Phytologia 47: 318. 1981.

#### Key to species

**Table:** 

1	Leaf margins crenulate; leaves bifid at least on small stature shoots; leaf cell surface ornamentation lacking urceolate papillae over the cell junctions	**2**
–	Leaf margins entire, not crenulate; leaves undivided; leaf cell surface ornamentation with urceolate papillae over the cell junctions on the medial-basal cells of some or all leaves	**Brevianthus flavus subsp. flavus**
2	Small triangular underleaves present on small to medium-sized shoot sectors, absent from large shoots; leaf margin not hyaline; leaf apex always distinctly bifid; leaf margin crenulate by thickened cell walls, marginal cells similar in size to internal cells	***Brevianthus hypocanthidium* M.A.M.Renner & J.J.Engel**
–	Underleaves entirely absent; leaf margin hyaline; leaf apex bifid on small leaves but undivided on large leaves, leaf margin crenulate by bulging cells, marginal cells smaller than internal cells	**Brevianthus flavus subsp. crenulatus J.J.Engel**

**Table 1.** Characters differentiating *Brevianthus* taxa.

	Brevianthus flavus subsp. flavus	Brevianthus flavus subsp. crenulatus	*Brevianthus hypocanthidium*
Leaf margin	Entire	Crenulate by bulging cell lumen	Crenulate by thickened cell walls
Leaf marginal cells	Hyaline	Hyaline	Chlorophyllous
Underleaves	Absent	Absent	Present on small and medium sized shoot sectors
Leaf apex	Unlobed	Unlobed on large leaves, bifid on small leaves	Uniformly bifid
Leaf shape	Ovate-oblate	Ovate-oblate	Ovate-rotund
Leaf cell trigones	Triangular to bulging	Triangular to bulging	Block-like
Leaf cell surface ornamentation	Urceolate papillae over cell junctions of at least medial and basal cells of some leaves on each shoot	Without urceolate papillae over cell junctions	Without urceolate papillae over cell junctions

### Brevianthus hypocanthidium

Taxon classificationPlantaeJungermannialesBrevianthaceae

M.A.M.Renner & J.J.Engel sp. nov.

Figures 1 , 2

#### Diagnosis.

Distinguished from both subspecies of *Brevianthus flavus* by the triangular underleaves produced on small- to medium-sized shoot sectors, the consistently but shallowly bilobed leaves, the crenulate leaf margins formed by heavily thickened exterior cell walls, and the chlorophyllous marginal leaf cells similar in size to the medial cells.

#### Type.

New Caledonia, Province Sud, Mont Kouakoué, slightly west of base camp at helicopter landing site, without date, *E.A. Brown 2006/17*, holotype: NOU; isotypes: NSW, F.

#### Description.

Plants closely prostrate, creeping, shoots sinuous, dull whitish green, opaque, water repellent, axes cylindrical, vermiform, to 2 mm wide and 30 mm long, the axes slightly laterally flattened. Branches sporadic, lateral-intercalary. Stolons and flagellae absent. Stems wiry, narrow for plant size, densely papillose, cortical cells oblong, all walls heavily thickened, in cross section 7–8 cells high, cortex undifferentiated, cells same size as medulla, with massive, nodular thickenings either confluent or separated by short stretches of unthickened primary wall. Rhizoids scattered on ventral side of stem, colourless, non-septate, tips often branched. Leaf insertion strongly succubous, nearly horizontal at postical end, not recurved at antical end, extending to stem mid-line on ventral side of large, but not small shoots, not extending to dorsal stem midline, leaving 1–2 cell rows leaf-free. Leaves strongly dorsally assurgent, not connivent over the dorsal stem surface, the axis appearing channeled in dorsal view with the stem partly or completely visible, the leaves unistratose throughout, densely imbricate, concave, ovate-rotund, lacking a hyaline border; apex shallowly but distinctly bifid; margins crenulate by thickened cell walls; dorsal margin rounded, not or slightly decurrent, ventral margin rounded, the base weakly auriculate on small leaves and not auriculate on large leaves, not overlapping the ventral stem surface, not totally obscuring the stem in ventral view. Leaf cells not tiered, polygonal but typically hexagonal, isodiametric, with massive coarse, nodular trigones, confluent or separated by narrow stretches of unthickened primary wall, primary walls visible within trigones, 39–50 µm diameter; marginal cells thick walled, external wall heavily thickened, especially medially, trigones coarse, not confluent, consistently separated by unthickened primary wall, lumena not reduced, cells only slightly smaller than median cells, quadrate, 40–50 µm long and 27–36 µm wide, long axis parallel with margin. Intramarginal cells on abaxial surface covered with dense circular and confluent to bacilliform anastomosing ornamentation continuous over cell junctions; urceolate to clavate ‘papillae’ over cell junctions absent. Cells, both marginal and intramarginal, on adaxial surface with similar ornamentation, comprised circular and confluent to weakly anastomosing ornamentation. Underleaves present on small- to medium-sized shoot sectors, triangular, 4–6 cells wide at base and 8–9 cell tiers high, apex acute, formed by a single cell; 230–300 µm long by 9–140 µm wide at base, margins crenulate; ventral merophyte 0–2 cells wide. Asexual reproduction by leaf-borne regenerants arising from the adaxial leaf surface.

Sexual structures not seen.

#### Etymology.

hypocanthidium: υπο- hypo-, below; αχανθα- acantha (f.) spine, thorn, prickle; -ιδιον -idion, a diminutive suffix.

#### Distribution and ecology.

So far as known, endemic to New Caledonia. The type collection occurred on a ridge bearing forest 3 m tall with open canopy and high light at ground level, where it grew with *Schistochila vitreocincta* (Herzog) X.-L.He & Glenny at the base of the trunk on a ‘mostly dead’ *Leucopogon* R.Br. The Schuster specimen occurred in an open, disturbed (old burn) *Dacrydium araucarioides* Brong. & Gris-*Callitropsis* Oerst. scrub.

#### Recognition.

The genus *Brevianthus* is highly distinctive among leafy-liverworts in the white or nearly white, water-repellent, cylindrical shoots with dorsally assurgent and succubously inserted leaves and no or inconspicuous underleaves, and scattered rhizoids. The shoots are typically sinuous in growth, either down or across the substrate, and lay closely appressed to it. They do not often overlap one another. This combination of macro-morphological characters facilitates field identification.

The three *Brevianthus* taxa recognized here all share these features, and are similar in their gross morphology. They differ primarily in micromorphological, microstructural, and anatomical details. However, characters vary in their manifestation with the stage of shoot stature and maturity, such that diagnostic differences must be sought within shoots of the appropriate age or size.

**Figure 1.**
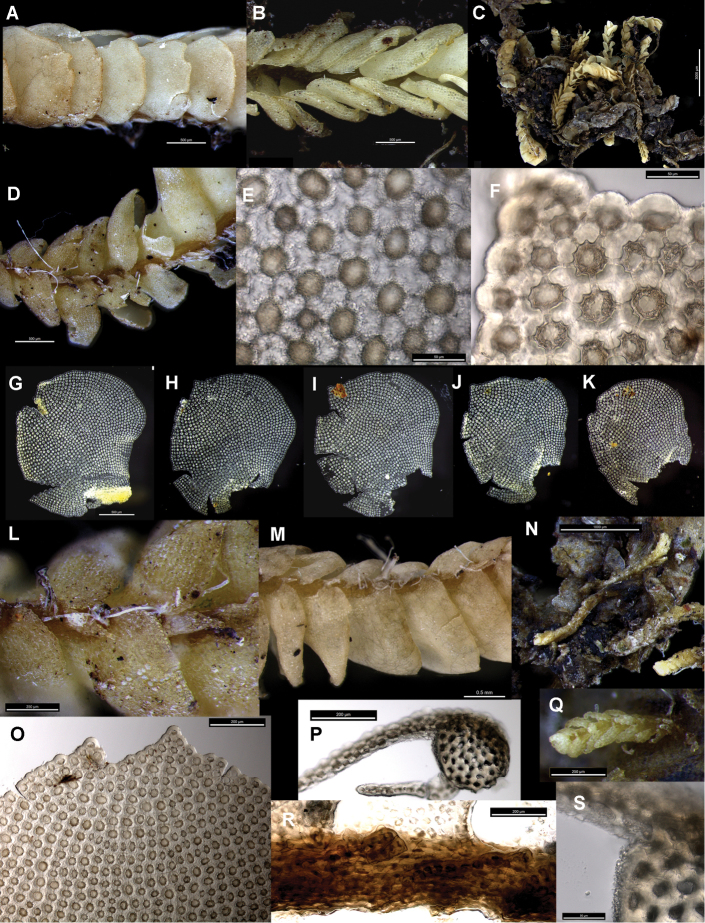
*Brevianthus hypocanthidium*: **A** lateral view of shoot **B** dorsal view of shoot **C** habitus **D** ventral view of shoot **E** abaxial leaf cell surface **F** cell margin at leaf apex, one leaf-lobe featured **G–K** leaves dissected and flattened **L** underleaves **M** ventral view of shoot showing underleaf absence from largest shoot sector (middle of image) **N** regenerants on old shoot sector **O** leaf apex **P** stem transverse section **Q** ventral view of regenerant attached to adaxial side of single cell at shoot base, showing variable expression of triangular underleaves on distinct ventral merophyte **R** lateral view of stem, with apex to left, leaves removed showing linear succubous insertion, J-shaped at ventral end, note cells of stem insertion projecting ventrally **S** transverse section of stem showing dense ornamentation on dorsal stem surface. All from NSW791547. Scale: 500 µm (**A, B, D, G–K, M**); 3000 µm (**C**); 50 µm (**E, F, S**); 200 µm (**L, O, P, R**); 1000 µm (**N**); 250 µm (**Q**).

The triangular underleaves found only in *Brevianthus hypocanthidium* (Fig. 1L) are a case in point. Not only are these partly obscured by adjacent leaves, they are produced only on small and medium sized shoot sectors. They are absent from the largest stature shoot sectors. As such, they are inconsistently present along a shoot, and may be entirely absent if the shoot examined is uniformly large. The other two *Brevianthus* taxa never produce underleaves, regardless of shoot stature.

Characters of the leaf apex and margins are useful in distinguishing the taxa of *Brevianthus*. The leaf apex of *Brevianthus hypocanthidium* (Fig. 1O) is shallowly but distinctly bifid, and this is a consistent feature of leaves of all sizes, though on the smallest leaves of leaf-borne propagules this is obscure. In Brevianthus flavus subsp. crenulatus small leaves are bifid (Fig. 5O, P), while medium and large leaves have an undivided apex (Fig. 5G–L). In Brevianthus flavus subsp. flavus the leaf apex is always undivided and entire (Fig. 3E–J).

The leaf margin provides several diagnostic differences between the three taxa that are of more consistent manifestation. In *Brevianthus hypocanthidium* the leaf margin (Fig. 1F) is crenulate by virtue of its heavily thickened exterior cell wall, and the marginal leaf cells are chlorophyllous and similar in size to the medial cells. In Brevianthus flavus subsp. crenulatus the leaf margin (Fig. 5F) is crenulate by virtue of bulging marginal cell lumena, and the marginal cells are colourless and smaller than medial cells. In Brevianthus flavus subsp. flavus the leaf margin (Fig. 3L) is entire, the marginal cells are again colourless and smaller than medial cells.

Trigones in leaf-cells differ between species. In *Brevianthus hypocanthidium* (Fig. 1E) they are block-like and angular with truncate ends and straight sides. In both subspecies of *Brevianthus flavus* (Figs 3L, 5F) they are coarse to bulging but with curved sides, and never as large or angular as observed in *Brevianthus hypocanthidium*.

**Figure 2.**
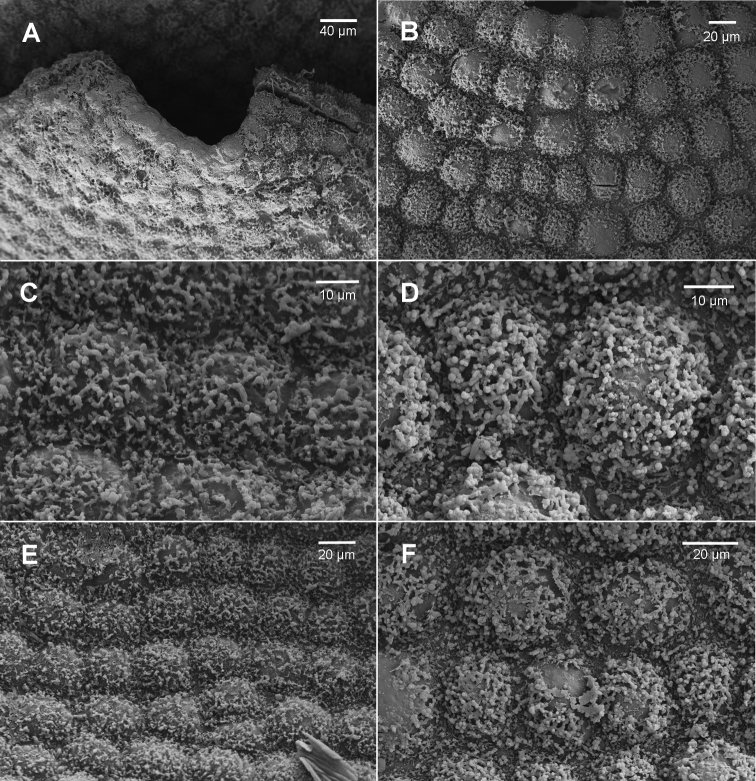
*Brevianthus hypocanthidium*: **A** leaf apex **B** cells at base of sinus on abaxial leaf surface **C** abaxial leaf surface at mid-leaf **D** abaxial leaf surface scale **E** adaxial leaf surface scale **F** adaxial leaf surface detail scale. All from NSW791547. Scale: 40 µm (**A**); 20 µm (**B, E, F**); 10 µm (**C, D**).

**Figure 3.**
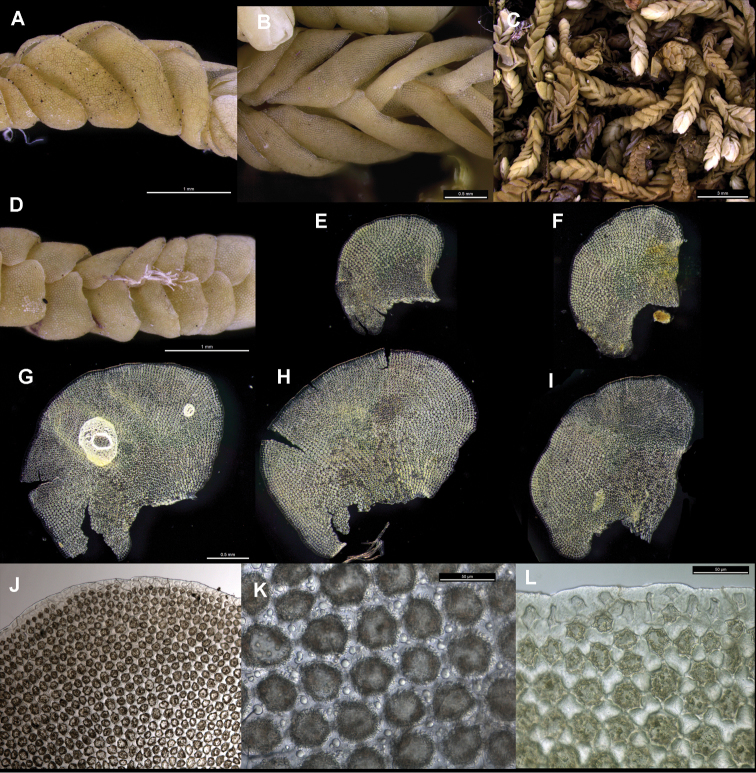
Brevianthus flavus subsp. flavus: **A** lateral view of shoot **B** dorsal view of shoot **C** habitus **D** ventral view of shoot **E–I** leaves dissected and flattened **J** leaf apex **K** abaxial leaf cell surface **L** leaf margin at apex. All from NSW892112. Scale: 500 µm (**A, B, E–I**); 3000 µm (**C**); 1000 µm (**D**); 200 µm (**J**); 50 µm (**K, L**).

**Figure 4.**
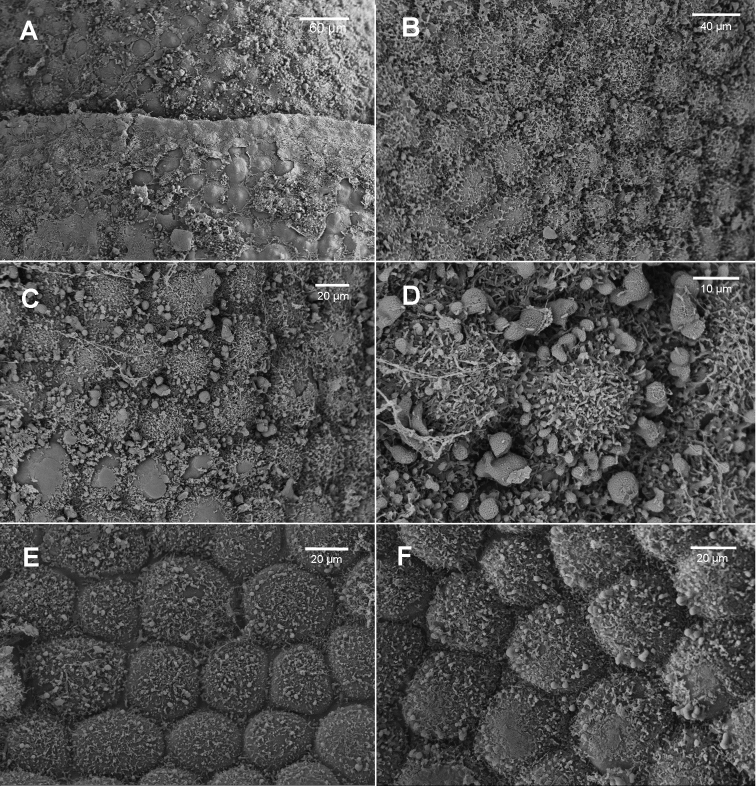
Brevianthus flavus subsp. flavus: **A** leaf apex scale **B** cells below apex on abaxial leaf surface scale **C** abaxial leaf surface at mid-leaf scale **D** abaxial leaf surface scale **E** adaxial leaf surface **F** adaxial leaf surface detail. All from NSW89112. Scale: 60 µm (**A**); 40 µm (**B**); 20 µm (**C, F**); 10 µm (**D**).

**Figure 5.**
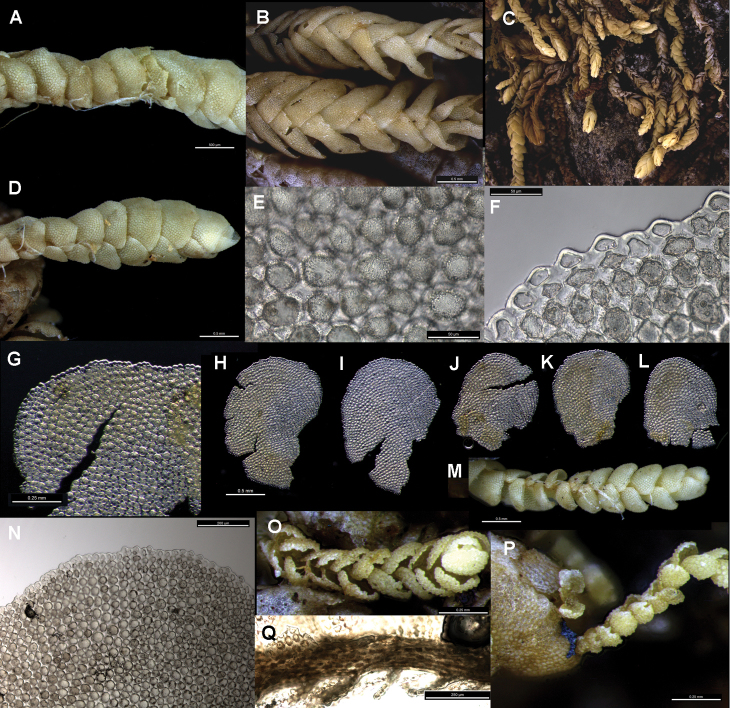
Brevianthus flavus subsp. crenulatus: **A** lateral view of shoot **B** dorsal view of shoot **C** habitus **D** ventral view of shoot **E** abaxial leaf cell surface **F** cell margin at leaf apex, one leaf-lobe featured **G** leaf showing crenulate hyaline cells prominent on dorsal margin **H–L** leaves dissected and flattened **M** ventral view of shoot **N** leaf apex **O** dorsal view of regenerant showing bulging cells and bifid leaf apices **P** lateral view of regenerant attached to adaxial side of single cell near the leaf margin at shoot base **Q** lateral view of stem, apex to left. All from NSW745726. Scale: 500 µm (**A, B, D, H–M**); 3000 µm (**C**); 50 µm (**E, F**); 250 µm (**G, O, P, Q**); 200 µm (**N**).

**Figure 6.**
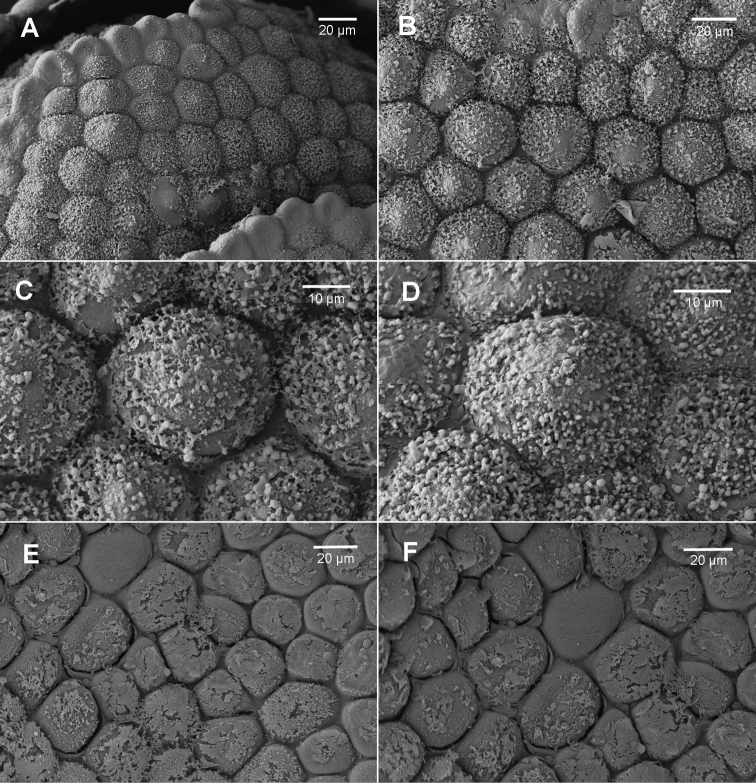
Brevianthus flavus subsp. crenulatus: **A** leaf apex **B** cells below apex on abaxial leaf surface **C, D** abaxial leaf surface at mid-leaf **E, F** adaxial leaf surface. All from NSW745726. Scale: 20 µm (**A, B, E, F**); 10 µm (**C, D**).

Leaf surface ornamentation may exhibit species-specific differences though there is intra-individual variation; our interpretation, however, may suffer from the relatively small number of observations we have made via SEM. Individuals of Brevianthus flavus subsp. flavus (Figs 3K; 4C, D) possess urceolate to clavate ‘papillae’ over the cell junctions on the abaxial leaf surface, at least between cells in the median-basal to basal portions of leaves, and at least sporadically on leaves along a single shoot. These ‘papillae’ have not been observed in individuals of Brevianthus flavus subsp. crenulatus (Figs 5E; 6C, D) or the type of *Brevianthus hypocanthidium* (Figs 1E; 2C, D). Parts of these structures are removable with chloroform, providing evidence that they partly consist of surface waxes (Heinrichs and Reiner-Drehwald (2012)).

Other leaf characters differentiate the taxa. The interstices between cells also appears to exhibit species-specific differences. In Brevianthus flavus subsp. crenulatus (Fig. 6) leaf cell junctions appear recessed within the leaf such that the upper and lower parts of the cell appear surrounded by a narrow trench, which is less pronounced or absent in both *Brevianthus hypocanthidium* (Fig. 2) and Brevianthus flavus subsp. flavus (Fig. 4).

Leaf shape, orientation and imbrication also differ. In both Brevianthus flavus subsp. crenulatus (Fig. 5D, M) and Brevianthus flavus subsp. flavus (Fig. 3D) the leaves are imbricate over the ventral stem surface, obscuring stem tissue in ventral view, while in *Brevianthus hypocanthidium* (Fig. 1D, L, M) the ventral stem surface is often partially visible between the leaves. The leaves of *Brevianthus hypocanthidium* (Fig. 1A, G–K) are ovate-rotund, and when viewed *in situ* laterally, have their antical margin orientated more or less perpendicular to the stem. Brevianthus flavus subsp. flavus (Fig. 1A, E–I) has ovate to oblate leaves whose antical margin is inclined in lateral view, with the lowest part of the margin closest to the shoot apex. The same is true of Brevianthus flavus subsp. crenulatus (Fig. 5A, H–L) though the angle of inclination is not so steep.

#### Conservation status.

That *Brevianthus hypocanthidium* is known from two gatherings precludes inference of its likely distribution and abundance, we therefore recommend the species be considered Data Deficient.

#### Additional specimen examined.

New Caledonia, Montagne des Sources, above St. Louis: Pic Buse and vicinity, 650–750 m, *R.M. Schuster 57820* (F).

### Brevianthus flavus (Grolle) J. J. Engel & R. M. Schust. subsp. flavus

Taxon classificationPlantaeJungermannialesBrevianthaceae

Phytologia 47: 318. 1981

Figures 3 , 4

Basionym: Jackiella flava Grolle, Journal of the Hattori Botanical Laboratory 33: 222. 1970.

#### Type.

Tasmania, Recherche Bay, Catamaran, 14 Jan 1911, *W.A. Weymouth 1232* as *Jamesoniella occlusa*, holotype: NY.

For a full description of *Brevianthus flavus* see Engel and Schuster (1982).

#### Distribution and ecology.

Brevianthus flavus subsp. flavus is widespread on the wetter western, south-western and southern sectors of Tasmania, between 300 and 900 m altitude where it occurs in a wide variety of habitat types encompassed by this altitudinal range. *Brevianthus flavus* occurs within or on the margins of a wide variety of forest types, for example riparian scrub dominated by *Leptospermum* J.R.Forst. & G.Forst., *Acacia* Mill. and *Banksia* J.R.Forst. & G.Forst., with dense thicket of *Bauera* Banks ex Andrews on alluvial terrace in a gully with south-easterly aspect at Condominium Creek, or montane forest of *Nothofagus cunninghamii* (Hook.) Oerst., *Eucryphia* Cav., *Arthrotaxis* Endl., *Atherosperma* Labill. and *Richea dracophylla* R.Br. with broken canopy to 8 m tall on a ridge with southerly aspect on Mt Dundas. The species also occurs in subalpine and alpine scrub such as that dominated by *Eucalyptus* L’Hér., *Sprengelia* Sm., *Leucopogon*, *Banksia*, *Orites* R.Br. and *Gymnoschoenus* Nees as at Mount Eliza, and dominated by *Astelia* Banks & Sol. ex R.Br., *Richea* and *Nothofagus* Blume at Mount Hesperus. *Brevianthus flavus* is for most part an epiphyte on tree trunks, including *Banksia*, *Lagarostrobos* Quinn, and *Nothofagus*. It sporadically occurs as a lithophyte on rock outcrops where it may inhabit crevices or sides sheltered by surrounding vegetation. It co-occurs with a wide variety of species, including *Acromastigum cavifolium* R.M.Schust., *Frullania* Raddi spp., *Heteroscyphus* Schiffn. spp., *Radula multiamentula* E.A.Hodgs. on rocks; *Bazzania* Gray, *Schistochila* Dumot. spp., *Lepidolaena brachyclada* (Lehm. ex Lehm.) Trevis., *Lepidozia ulothrix* (Schwägr.) Lindenb., *Schistochila pinnatifolia* (Hook.) Trevis., *Radula* spp., *Acromastigum cavifolium*, *Acrobolbus cinerascens* (Lehm. & Lindenb.) Schiffn., *Acrobolbus ochrophyllus* (Hook.f. & Taylor) R.M.Schust., *Frullania* etc on trunks of *Nothofagus cunninghamii*.

#### Conservation status.

Brevianthus flavus subsp. flavus appears widely distributed in western and southern Tasmania, however collecting and survey effort to date provides insufficient basis for an accurate appraisal of the species’ threat status. We therefore recommend Brevianthus flavus subsp. flavus be regarded as Data Deficient pending a more informed assessment.

#### Specimens examined.

Australia, Tasmania, West: Highway B28, east of Mt Murchison, Quinn Creek, 41°50'S, 145°37'E, 600 m, 20 Feb 1998, *J.E. Braggins 98064B*, AK255753; West Coast: Mount Dundas Regional Reserve, Mount Dundas, track to summit from south, 41°54'32"S, 145°28’3 2"E, 845 m, *M.A.M. Renner 6008 & E.A. Brown*, NSW855958; South West: Serpentine River valley, south of the Gordon River, just below the dam, 42°47'S, 145°57'E, 300m, 18 Feb 1998, *J.E. Braggins 98052A*, AK255728; South West Conservation Area: Mount Eliza, Condominium Creek, 42°57'22"S, 146°21'56"E, 350 m, 23 Jan 2012, *M.A.M. Renner 5927 & E.A.Brown*, NSW895251; Mount Eliza, unnamed catchment S of Mount Anne track, 42°57'45"S, 146°23'32"E, 860 m, 22 Jan 2012, *M.A.M. Renner 5898 & E.A.Brown*, NSW892112; Arthur Range, Mount Hesperus, S of track to summit at top of hill, 43°06'41"S, 146°13'9"E, 820 m, 24 Jan 2012, *M.A.M. Renner 5958*, NSW880771.

### Brevianthus flavus subsp. crenulatus

Taxon classificationPlantaeJungermannialesBrevianthaceae

J.J.Engel, Nova Hedwigia 93: 406. 2011

#### Type.

New Zealand, South Is., Westland Prov., Lake Kaniere Scenic Reserve, Lake Kaniere Rd, 125 m, *J.J. Engel 24815, M.J. von Konrat & J.E. Braggins*. holotype: F; isotype: CHR.

#### Distribution and ecology.

Brevianthus flavus subsp. crenulatus exhibits a discontinuous distribution within New Zealand's, cool hyper-humid forest environments from Waipoua Forest in western Northland southward at least as far as Blackball on the West Coast of the South Island. Brevianthus flavus subsp. crenulatus is often, though not exclusively, associated with forests including *Lepidothamnus intermedius* (Kirk) Quinn or *Manoao colensoi* (Hook.) Molloy, where these occur in podocarp-broadleaf forests, as at the summits of Hirakimata (Mount Hobson) on Aotea (Great Barrier Island, M.A.M. Renner *pers. obs.*) and Mount Rowe; or in podocarp-beech forest as at Craigieburn. Brevianthus flavus subsp. crenulatus is typically a trunk epiphyte, but may occur as a lithophyte, as at the head of the Croesus Track, near Blackball.

In Northland Brevianthus flavus subsp. crenulatus may be a common and even dominant component of epiphytic communities on the southern side of *Metrosideros robusta* A.Cunn. and *Agathis australis* (D.Don) Loudon trunks in open *Agathis australis* ricker forest on clearing edges, where it occurs with *Dendromastigophora flagellifera* (Hook.) R.M.Schust., *Lepicolea attenuata* (Mitt.) Steph., *Lopholejeunea colensoi* Steph., *Radula pseudoscripta* M.A.M.Renner, *Heteroscyphus menziesii* (Mitt.) J.J.Engel, and *Hymenophyllum armstrongii* (Baker) Kirk. Despite its often luxuriant growth in these situations, no fertile material has yet been observed.

From the Auckland Region southward Brevianthus flavus subsp. crenulatus tends to be an epiphyte on trunks of *Lepidothamnus intermedius*, *Manoao colensoi* and sometimes Nothofagus solandri (Hook. f.) Oerst. var. cliffortioides (Dippel) Poole, where these grow in mixed podocarp-beech and podocarp-broadleaf forests, such as the *Lepidothamnus intermedius*, *Dacrydium cuppressinum* Sol ex G.Forst., Phyllocladus aff. alpinus Hook.f., *Weinmannia silvicola* L.f., *Quintinia serrata* A.Cunn., *Pseudopanax discolor* (Kirk) Harms forest on boggy ground on the summit ridge of Mt Rowe, here Brevianthus flavus subsp. crenulatus grew in association with *Lepicolea scolopendra* (Hook.) Dumort. ex Trevis., *Lepidozia microphylla* (Hook.) Lindenb., *Heteroscyphus* sp., *Acrochila biserialis* (Lehm. & Lindenb.) Grolle, *Acromastigum cavifolium*, *Schistochila tuloides* (Hook.f. & Taylor) Steph., *Radula multiamentula*, and *Thysananthus anguiformis* (Hook.f. & Taylor) Taylor ex Gottsche, Lindenb. & Nees.

At the head of the Croesus track Brevianthus flavus subsp. crenulatus grew on vertical granite of bluffs overhanging the start of the Croesus track, with *Heteroscyphus menziesii*, *Radula multiamentula*, *Acrobolbus epiphyticus* (Colenso) Briscoe, *Herbertus* Gray, *Acromastigum anisostomum* (Lehm. & Lindenb.) A.Evans, *Frullania* sp., *Lepidozia* (Dumort.) Dumort. spp. and *Hymenophyllum armstrongii*.

At Craigieburn Road on the West Coast of the South Island, Brevianthus flavus subsp. crenulatus is an occasional corticol on *Manoao colensoi* trunks in low forest with uneven, broken canopy comprised of *Leptospermum scoparium* J.R.Forst. & G.Forst. with Nothofagus solandri var. cliffortioides, *Nothofagus menziesii* (Hook.f.) Oerst., and emergent *Manoao colensoi*, on saturated soil of an old alluvial terrace. Here the species was common on *Manoao* trunks, occasional on Nothofagus solandri var. cliffortioides, but apparently absent from trunks of *Nothofagus menziesii*, and grew in association with *Acrochila biserialis*, *Acromastigum cavifolium*, *Heteroscyphus menziesii*, *Heteroscyphus* sp., *Schistochila tuloides*, *Radula multiamentula*, *Radula tasmanica* Steph., *Frullania ptychantha* Mont., *Frullania* sp., *Macromitrium longipes* (Hook.) Schwägr., *Dicnemon calycinum* (Hook.) Schwägr., and *Hymenophyllum armstrongii*.

#### Conservation status.

Brevianthus flavus subsp. crenulatus was listed as Naturally Uncommon, with qualifier ‘Sparse’ by the New Zealand Department of Conservation's, Threat listing Bryophyte specialist panel (Glenny et al. 2014).

#### Specimens examined.

New Zealand, North Island: Western Northland Ecological Region, Tutamoe Ecological District, Waipoua Forest, Tarahoka clearing, 35°37'S, 173°33'E, 380 m, 16 Oct 2000, *M.A.M. Renner 00/125*, AK280186; Tutamoe Ecological District, Waipoua Forest, lookout loop, Toatoa Grove, c. 280m, 35°40'30"S, 173°33'46"E, 21 Feb 1990, *J.E. Braggins 90/42*, AK325216; Waipoua Forest, track to Te Matua Ngahere, 35°36'S, 173°31'E, ca. 340 m, 1997, *J.J. Engel 22543* (F); Thames Ecological District, ridge NW of Mount Rowe on track to summit, 37°02'16"S, 175°40'19"E, 720 m, 14 Feb 2007, *M.A.M. Renner 2548*, NSW745726; Coromandel Ecological Region, Thames Ecological District, Ridge NW of Mt Rowe, track to Mt Rowe, 37°02'16"S, 175°40'19"E, 720m, 14 Feb 2007, *M.A.M. Renner 2535*, AK298528; Coromandel Ecological Region, Thames Ecological District, Ridge NW of Mt Rowe, track to Mt Rowe, 720 m, 37°02'16"S, 175°40'19"E, 14 Feb 2007, *M.A.M. Renner 2618*, AK299749; Coromandel State Forest Park, summit of Table Mt., 37°03'S, 175°40'E, 835 m, 1997, *J.J. Engel 22381* (F); South Island: North Westland Ecological Region, Blackball Ecological District, Craigieburn, Craigieburn Road east, 220 m, 42°13'43"S, 171°37'30"E, 06 Dec 2000, *J.E. Braggins*, AK287110; North Westland Ecological Region, Blackball Ecological District, Craigieburn, Craigieburn Road east, 190 m, 42°14'S, 171°38'E, 28 Mar 2001, *M.A.M. Renner 01/200*, AK280202; North Westland Ecological Region, Maimai Ecological District, Cragieburn Road, near Doolan Creek headwaters, west of Atarau Road, some 400m south of *Pinus* plantation in dense low mixed podocarp/*Nothofagus* forest, 205 m, 42°14'S, 171°38'E, 30 Apr 2003, *Y. Qiu NZ03115 & J.E. Braggins*, AK283714; North Westland Ecological Region, Blackball Ecological District, Paparoa Range, Croesus track, Blackball Road end, 42°20'S, 171°24'E, 330m, 26 Mar 2001, *M.A.M. Renner s.n.*, AK280201.

## Discussion

The presence of underleaves in *Brevianthus hypocanthidium* reduces the morphological distance between this isolated genus and more typical Lophocoleaceae species including those belonging to *Tetracymbaliella*, which was shown to be sister to *Brevianthus* in the analysis of He-Nygren et al. (2006). The retention of at least partial underleaf production in *Brevianthus hypocanthidium* is probably plesiomorpic and the complete absence of underleaf production in *Brevianthus flavus* probably derived. The variable production of underleaves and a ventral merophyte by mature gametophyte shoots in *Brevianthus hypocanthidium* is unusual within species of the Lophocoleaceae-Plagiochilaceae complex, and perhaps all Jungermanniopsida Stotler & Crand.-Stotl. Only in broad phylogenetic terms is a parallel seen, in that across the Jungermanniales Hedw. as a whole, as stature of the lateral merophytes increases so does anisophylly, which is at the expense of the ventral merophyte (Schuster 1966).

In Lophocoleaceae the narrow triangular underleaf is unusual in showing no evidence of lobing. While well-developed and unlobed underleaves are known within the Lophocoleaceae, as in *Chiloscyphus austrigenus* (Hook.f. et Taylor) R.M.Schust. et J.J.Engel (Engel 2010), and many species of *Heteroscyphus* (J.J.Engel unpublished data); all of these taxa have a wide ventral merophyte. Underleaves in most species of the family, however, are bilobed in all stages of expression. In the related Plagiochilaceae, whose species for the most part have reduced ventral merophytes, unlobed and ciliform underleaves are known, for example, in *Dinckleria fruticella* (Hook.f. & Taylor) J.J.Engel & Heinrichs. In both *Dinckleria fruticella* and *Brevianthus hypocanthidium* the underleaves are probably derivatives of a reduced ventral merophyte, whose abbreviated morphology reflects underlying developmental degeneracy.

What then of the variable underleaf production exhibited by *Brevianthus hypocanthidium*? That merophytes form continuous rows, two lateral and one ventral, all contributing stem and leaf tissue implies the leaf and underleaf insertions ought not exhibit overlap across shared cell rows along the stem. This generally holds across the Jungermanniopsida as a result of the orderly proliferation of cortical cells within each merophyte row.

Dorsal and ventral ‘leaf-free’ strips are readily reconciled via greater division of stem-producing derivatives of the merophyte initial. The converse, overlap of leaf and underleaf insertion lines across rows of cortical stem cells, is not so readily reconciled with a model of growth wherein each merophyte derivative contributes to discrete stem sectors.

**Figure 7.**
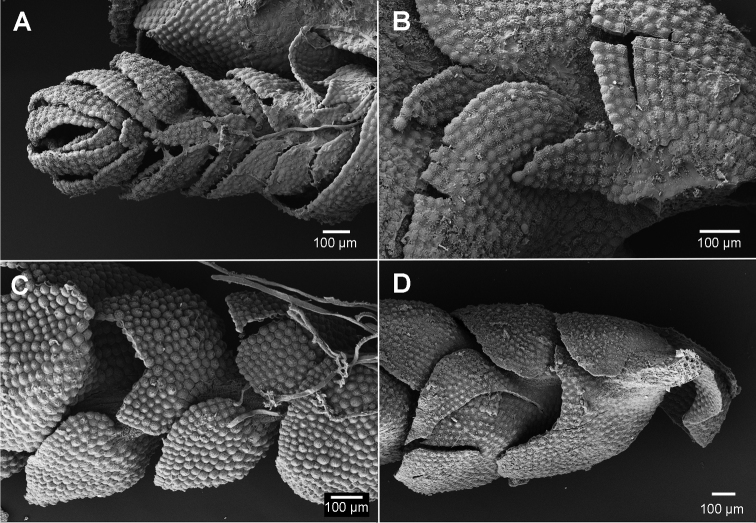
Ventral stem views. **A, B** *Brevianthus hypocanthidium* (NSW791547) **C** Brevianthus flavus subsp. crenulatus (NSW745726) **D** Brevianthus flavus subsp. flavus (NSW892112).

In small stature shoots of *Brevianthus hypocanthidium* the leaf insertion lines do not reach the ventral stem mid-line, leaving a row or two of ventral cortical cell rows leaf-free, onto which the underleaves are inserted. In small stature shoots the merophyte rows appear both continuous and non-overlapping, and their growth is compatible with conventional liverwort development (Fig. 7).

However, in medium-sized shoot sectors where underleaves are produced, the insertion lines of the two lateral merophytes reach the ventral stem midline. The underleaf insertion line is completely overlapped by the insertion line of the non-adjacent leaf (Figs 1L, M; 7B). This is incompatible with a conventional growth model involving three continuous and discrete merophyte rows. How might the underleaves come to be completely nested within the overlapping leaf insertion lines? One explanation could be that the ventral merophyte row is not straight but perhaps zig-zags between the leaf insertion lines. Observation of ventral cortical cell rows is hampered by their surface ornamentation, rhizoids, and their overlap by both underleaves and lateral leaves. We have not undertaken destructive sampling of underleaf-bearing shoots to confirm this due to the relative paucity of the type material. So while we cannot categorically exclude this possibility by direct observation of cortical cell rows, it fails to explain why underleaves are not produced on large shoots.

The stature-correlated pattern of underleaf production in *Brevianthus hypocanthidium* provides clues to development, and there are a number of observations a developmental model must explain:

1. Shoot stature increases and underleaf size remains constant on small and medium sized shoots.
2. Underleaves are absent from large shoot sectors.
3. Lateral leaf insertion does not reach the ventral stem midline on small shoots, therefore a discrete and continuous merophyte row is present.
4. Lateral leaf insertion reaches the ventral stem midline on medium sized and large shoots, therefore a discrete and continuous merophyte row is lacking.
5. Underleaf insertion is completely overlapped by the leaf insertion lines on medium-sized shoots.
6. Underleaf position relative to the adjacent leaf on medium-sized shoots varies. Sometimes the underleaf is next to the adjacent leaf on a continuation of the same insertion line. At other times the underleaf is behind and a little more apical in position to the leaf on a separate line of insertion that overlaps the insertions of the adjacent leaf as well as the opposing leaf.

Assumption of a helical segmentation sequence seems reasonable given the apparent invariance across the Jungermanniales and its manifestation on at least small and medium-sized shoots, as evidenced by the sequence of merophytes. The most unusual and counter-intuitive features are the presence of underleaves when lateral leaf insertion lines reach the ventral stem mid-line, and the absence of underleaves from the larger shoots.

Here we posit a competitive model of shoot growth to explain the five observations above, wherein merophytes vie for occupancy of the mature shoot. An increase in shoot stature can be achieved by an increase in stature of the lateral merophytes only, or shoot stature can be increased by increasing the size of the lateral merophytes at the expense of ventral merophyte stature (Schuster 1966).

In *Brevianthus hypocanthidium* both may contribute, and our competitive model combining changes in both lateral and ventral merophytes is postulated as follows. The stature of lateral merophytes increases with shoot stature, while the stature of ventral merophytes does not, resulting in a decrease in relative stature of the ventral merophyte. With reduced stature comes a reduction in relative vigour of ventral merophyte derivatives, resulting in proportionally fewer cell divisions particularly those that contribute to the stem cells. The derivative cells fail to occupy the complete length of the potential ventral stem surface, they become localized to the region of initial deposition only. In such cases the ‘vacant’ ventral stem surface is occupied by tissue derived from lateral merophytes. These lateral merophyte derivates carry their leaf insertion lines to the ventral stem midline. The ventral merophytes become “marooned” or isolated at the sites of deposition. Growth of opposite and adjacent lateral merophytes causes the ventral merophyte to appear both laterally displaced and enveloped by the lateral merophytes. Perhaps eventually the ventral merophyte initials lose vigour to the extent that no divisions resulting in leafy tissue are completed. Developmental studies, perhaps including selective sequential sectioning, might test this model when more material becomes available.

Perianths and bracts. In *Plagiochila* shoot stature increases prior to gynoecium production. A female bracteole is often produced, as may be underleaves associated with one or two of the subtending gyres. In some members of this genus increase in shoot stature results in the re-expression of leafy appendages on the ventral merophyte. The same ought be true in *Brevianthus hypocanthidium*, given that in *Brevianthus flavus* bracteoles are expressed in the two cycles of female bracts on gynoecium-bearing branches. These bracteoles are either broadly connate or free from the adjacent bracts (Engel and Schuster 1982). The brevity of the lateral intercalary branches upon which gynoecia are borne in this species precludes assessment of transformation from normal leafy shoots. Location of fertile material of *Brevianthus hypocanthidium* would confirm the consistency of gynoecium position and associated characters within the *Brevianthus* lineage.

## Supplementary Material

XML Treatment for Brevianthus

XML Treatment for Brevianthus hypocanthidium

XML Treatment for Brevianthus flavus (Grolle) J. J. Engel & R. M. Schust. subsp. flavus

XML Treatment for Brevianthus flavus subsp. crenulatus
